# A Highly Linear Ultra-Low-Area-and-Power CMOS Voltage-Controlled Oscillator for Autonomous Microsystems

**DOI:** 10.3390/mi15101193

**Published:** 2024-09-26

**Authors:** Javier de Mena Pacheco, Tomas Palacios, Marek Hempel, Marisa Lopez Vallejo

**Affiliations:** 1 IPTC, E.T.S.I. Telecomunicación, Universidad Politécnica de Madrid, 28040 Madrid, Spain; m.lopez.vallejo@upm.es; 2 MTL, Massachusetts Institute of Technology, Cambridge, MA 02139, USA; tpalacios@mit.edu; 3 Analog Devices, Inc., Wilmington, MA 01887, USA; marek.hempel@analog.com

**Keywords:** VCO, smart dust, linearity, ultra-low frequency

## Abstract

Voltage-controlled oscillators (VCOs) can be an excellent means of converting a magnitude into a readable value. However, their design becomes a real challenge for power-and-area-constrained applications, especially when a linear response is required. This paper presents a VCO for smart dust systems fabricated by 65 nm technology. It is designed to minimize leakage, limit high peak currents and provide an output whose frequency variation is linear with the input voltage, while allowing rail-to-rail input range swing. The oscillator occupies 592 $\mu m^{2}$, operates in a frequency range from 43 to 53 Hz and consumes a maximum average power of 210 pW at a supply voltage of 1 V and 4 pW at 0.3 V. In addition, the proposed VCO exhibits a quasi-linear response of frequency vs. supply voltage and temperature, allowing easy temperature compensation with complementary to absolute temperature (CTAT) voltage.

## 1. Introduction

Autonomous microsystems are systems that require no external power to operate and communicate for a given period of time, while having dimensions smaller than the diameter of a human hair (<100 μm). These microsystems enable new uses for electronics, made possible solely by their smaller size, such as sensing in confined spaces, like microfluidic channels [1] or the human body. However, their main disadvantage is that their tiny size results in a very limited power budget and reduced functionality.

Analog-to-digital conversion is one of the first and most important steps to be performed in any system working with signals from the physical world. Traditional ADCs perform this conversion by comparing the signal’s amplitude with respect to a given reference. Still, they tend to be very area- and power-hungry for applications where these two characteristics are severely constrained. In a microsystems context, digitization can be achieved in the time domain by using voltage-controlled oscillators (VCOs), where the input signal is converted to a modulated frequency and the output can be easily digitized by measuring the frequency with counters. However, the main challenge with this type of conversion is to achieve a linear voltage–frequency response, since the resolution of the digitization is highly dependent on the linearity of the VCO. Many VCO-based converters [2] include calibration circuitry, such as look-up tables (LUTs) [3], to linearize their performance, but these take up considerable area and power.

On the other hand, since autonomous microsystems do not need to be fast, a common approach to reducing power consumption is to operate at very low frequencies [4]. However, this is neither easy nor trivial when the large passive components required to generate slow time constants are not affordable, due to area constraints. The most commonly used strategies for designing very slow oscillators within these constraints are based on the use of very low currents, such as those provided by the transistor gate leakage current [5,6] and subthreshold leakage current [7,8,9,10].

Many other systems, apart from autonomous microsystems, require to stay in *idle* state for very long periods when they enter sleep mode, to save power [5]. Most of the time, only a few subsystems remain active, and the wake-up timer is always one of them. Consequently, wake-up timers must consume very little energy, making ultra-low-frequency- and -power oscillators very suitable for these cases. It can also be interesting to put the system into hibernation for a longer or shorter period, depending on a certain magnitude or condition, so it is desirable to equip these oscillators with a simple mechanism to vary their frequency without significantly worsening the energy consumption.

Our target scenario is a special case of autonomous microsystem, the second generation of *MIT SynCells* [11], which includes a 50 μm × 50 μm Si chip that, among other tasks, interfaces the analog voltage coming from a sensor. Given the limited area and power, voltage-to-frequency conversion [12] emerges as an interesting way to perform digitization. In this paper, we propose a highly linear VCO with ultra-low area, power and frequency. It is important to note that no reported ADC comes close to meeting the very stringent constraints of working in the sub-nW power regime and cell size scale. The chip was designed and manufactured using 65 nm commercial CMOS technology. This technology provides a good compromise between a small transistor size, which directly translates into die area reduction and affordable variability, and short channel effects and matching issues, which mostly complicate the design of the analog blocks of the microsystem.

The main contributions of this work are as follows:

- Design of a 65 nm ultra-low frequency–area–power VCO with linearized response of the frequency vs. the control voltage.
- The proposed linearization method is applicable to other oscillator structures and any available supply range.
- Quasi-linear response of frequency vs. supply voltage and temperature, making it easy to achieve temperature compensation with a complementary to absolute temperature (CTAT) voltage supply type.

The paper is organized as follows. First, we introduce the microsystem in which the VCO will operate. Next, the delay element used in the oscillator is presented along with the constraints that drove the entire design process. The proposed VCO is then described and analyzed. Finally, the results are presented and some conclusions are drawn.

## 2. Overall System Description and Design Constraints

**Figure 1:**
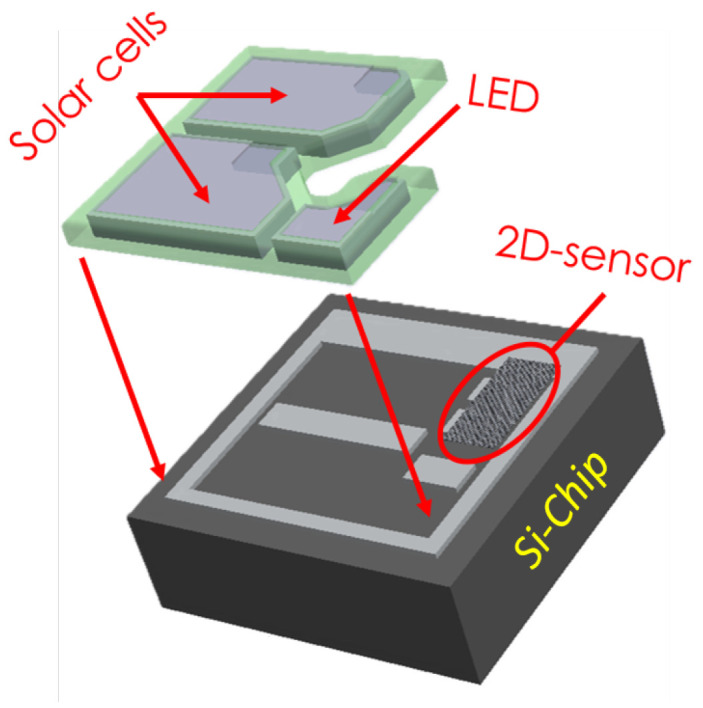
Integration scheme for the *SynCell*.

Figure 1 shows a 3-D representation of the integration of the targeted 50 × 50 $\mu m^{2}$ MIT microsystem for which this VCO was designed. The second generation of *SynCells* consists of two GaN solar cells connected in series for energy harvesting, a $MoS_{2}$-based sensor, a GaN LED for optical communication and a CMOS *Si*-chip that regulates the power generated by the solar cells and drives the LED while serving as the carrier for the rest of the microsystem elements. In this way, the information captured by the sensor is transmitted to the outside world through light pulses of varying frequency. The sensor is composed of two $MoS_{2}$-based devices set in a voltage divider configuration. One of them is passivated while the other one is sensitive to chemical exposure. This sensor setup converts the change of resistance in a voltage change that can swing from 0.2 $V_{dd}$ to 0.8 $V_{dd}$, depending on the chemical concentration. The VCO described in this paper converts the voltage from the sensor into a variable-frequency square-wave signal. The output of the VCO is then used by the LED driver to control the current flow to the LED, causing the LED to flash at a frequency proportional to the physical magnitude being measured. In this way, the sensor information is communicated to the receiver, which captures the light pulses with a CCD camera.

The extremely reduced size of the microsystem necessitates that the implemented solar cells are also very small (23 × 35 $\mu m^{2}$). Therefore, the generated power was particularly limited for this scenario. Before integrating the microsystem, a GaN solar cell was tested in-lab, to determine the power budget of the design. For the microsystem to be able to be powered on low-energetic light, such as ambiance light, a constrained power budget of only 200 nA was estimated. The LED requires at least 60 nA to become visible and the sensor can draw up to 100 nA, depending on the chemical concentration.

Due to its very weak nature, the microsystem’s power source can collapse if the required current exceeds certain limits, causing a critical voltage drop that will shut down the system. Therefore, a sub-nW VCO is highly desirable for the system. However, it is important not only to keep the average power consumption within affordable limits but also to monitor the transient power peaks carefully. Even if these peaks are not large enough to collapse the system, they can cause variations in the supply voltage that can affect the precision with which the system operates. Hence, peak currents in the order of nA are targeted for the VCO, so as not to interfere with the proper operation of the solar cells.

In the first in vitro application of the *SynCell*, the measured magnitude is a chemical compound known as putrescine, which is a diamine present in all organisms [13]. In meat or fish, its concentration increases as decomposition begins, making it a natural bioindicator of food quality [14]. Additionally, putrescine can be an indicator of cell proliferation and cell growth [15]. In this way, it is particularly relevant in plants [16]. The absence of putrescine in plants is associated with an increase in both parasite and fungal populations.

As a result, autonomous tracking of putrescine concentrations in a very localized manner shows many beneficial use cases.

## 3. Sub-Threshold Leakage-Based Delay Element

Most oscillators designed for very slow frequencies currently use large R, L and C elements or frequency dividers that take up too much area. However, we can use the sub-threshold leakage currents of CMOS transistors to generate large time constants, together with a reduction in short circuit currents, by creating very steep slopes through positive feedback. This approach results in ultra-low power consumption while keeping the area under very low limits. To achieve this, we used the element proposed in [7] as a starting point for our voltage-controlled oscillator. However, when this element is scaled down, important modifications are required, to achieve ultra-low frequency–area–power goals.

**Figure 2:**
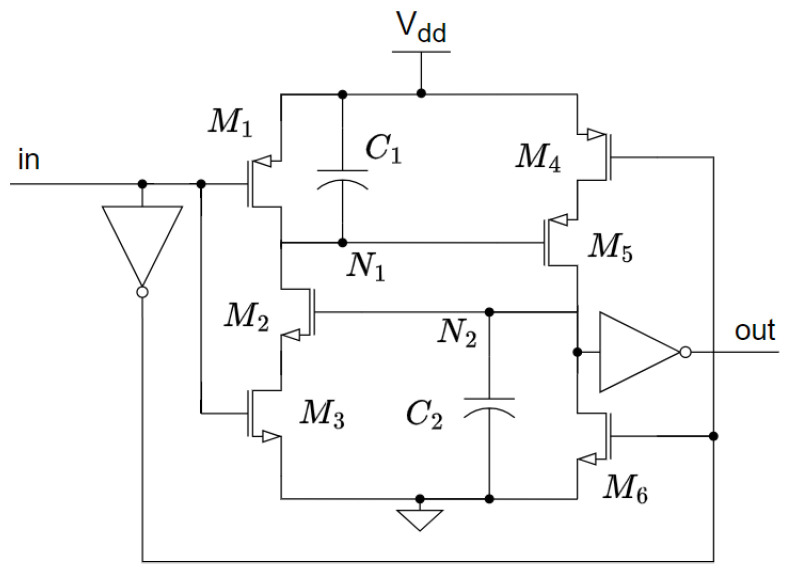
Leakage-based delay element.

The delay element [7] is based on a thyristor structure, as is shown in Figure 2. To explain how it works, let us assume that the input is low, which causes transistors $M_{1}$ and $M_{6}$ to be on and $M_{3}$ and $M_{4}$ to be off. This leads node $N_{1}$ to charge to $V_{dd}$ and $N_{2}$ to discharge to GND. Consequently, transistors $M_{2}$ and $M_{5}$ are off. A transition from low to high of the signal In is then assumed. $M_{1}$ and $M_{6}$ are now off while $M_{3}$ and $M_{4}$ are turned on. The leakage current of $M_{5}$ is then used to charge $C_{2}$ and the leakage current of $M_{2}$ discharges the negative terminal of $C_{1}$. When the voltage on $C_{2}$ becomes greater than the threshold voltage, $V_{TH,n}$, $M_{2}$ becomes conductive, fully discharging $N_{1}$, which exponentially increases the conductivity of $M_{5}$ and, thus, the voltage on $N_{2}$. This positive feedback loop ensures that the transition of the output node $N_{2}$ is slow when all transistors are in the sub-threshold region and fast when the transistors are conducting. Although the overall power consumption is very low, peak currents in the microampere range have been observed during the short period when the transistors are conducting. These currents represent an excessive power demand if energy harvesters were to power the system, and they must be significantly reduced.

Eventually, the delay provided by this circuit is given by

(1) $$t_{d,M_{5},C_{2}}=\frac{C_{2}}{I_{leak,M_{5}}-I_{leak,M_{6}}}\times V_{TH,n}$$

The full oscillator consists of *N* cascaded delay elements arranged and connected as a ring oscillator with a period *N*-times the delay of a single delay element.

At the same time, ref. [7] suggested a method to make the frequency controllable. By adding four transistors, the currents used to charge $C_{2}$ and discharge $C_{1}$ can be increased or decreased. However, their structure does not satisfy several design features that we consider critical for our target applications:

- The V-f response is highly non-linear; it follows an exponential equation, which results in a poor resolution when this VCO is used for time domain digitization.
- The power consumption grows exponentially with the control voltage, a critical drawback for ultra-low power systems.
- The input range does not support a rail-to-rail input swing, which is also necessary for time-domain digitization.
- Important current peaks, in the microampere range, were found in the structure presented in [7], which can cause some energy harvesters to collapse.

In this work, a highly linear VCO is presented, to overcome all these issues.

## 4. Proposed Structure

**Figure 3:**
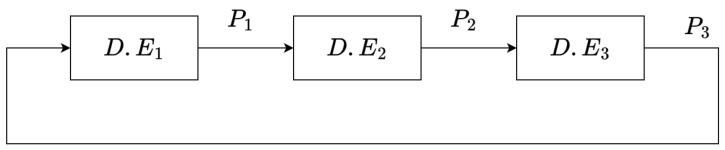
VCO composed of three delay elements.

**Figure 4:**
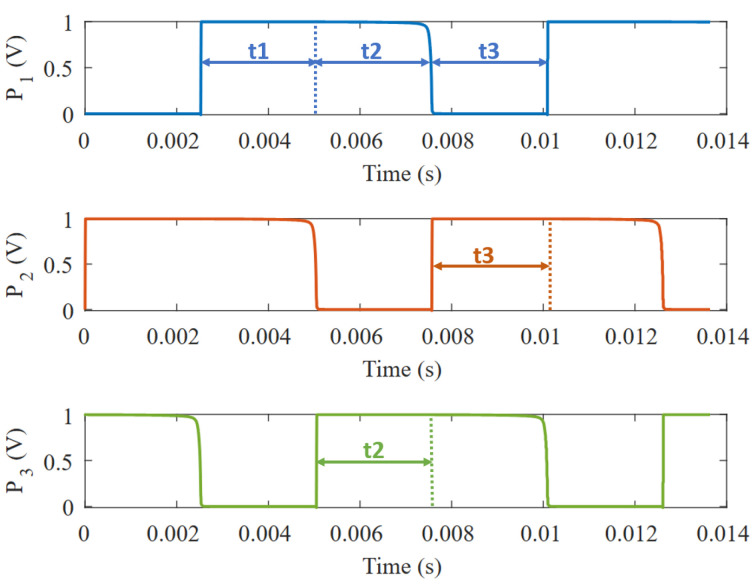
Transient simulation of the three phases of the VCO.

The proposed VCO follows a ring-oscillator structure with three delay elements connected in series, as shown in Figure 3. Consequently, the period is given by the addition of the three delays generated in the circuit, as illustrated in Figure 4.

**Figure 5:**
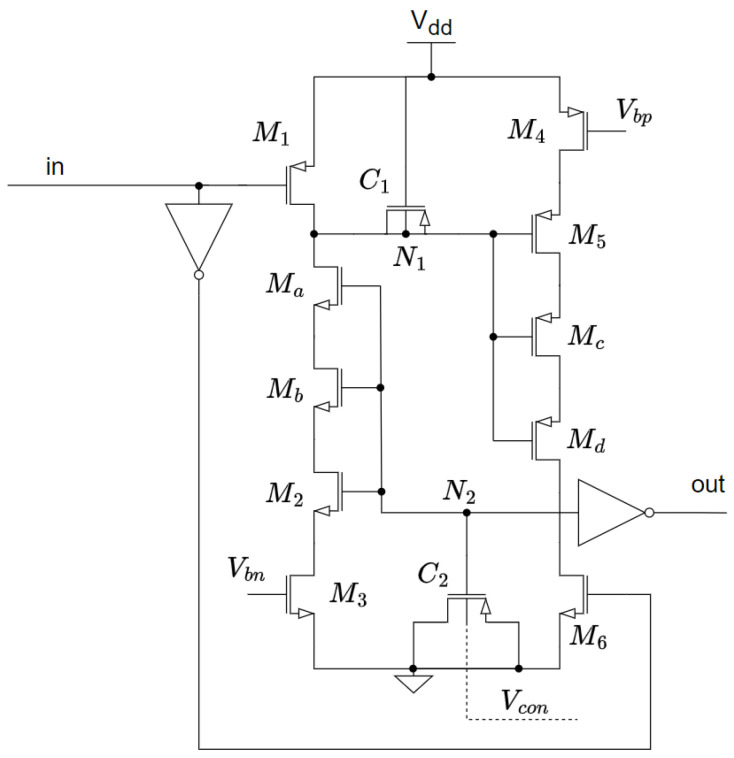
Proposed delay element with capacitance tuning as voltage control.

The thyristor-based delay element used to solve the aforementioned problems is shown in Figure 5. The main goals of this design were: (1) to provide an ultra-slow frequency with reduced area; (2) to reduce the peak power; and (3) to generate an output frequency that varies linearly with an input voltage, while having no significant impact on area or power consumption.

### 4.1. Large Time Constants with Nanometer Technologies

The best way to reduce the area of microsystem circuits is to use a smaller technology node. This simple solution, which directly benefits the size of the circuits, comes with many other drawbacks and challenges. One of them, as explained in [17], is that leakage currents become more significant with smaller technology nodes.

Since the goal of our circuit is to generate very low oscillation frequencies, to dissipate as little power as possible, we first looked at methods of modifying and controlling the leakage currents that play a key role in the oscillator.

In our case, the leakage currents for 65 nm technology are significantly higher than for the 180 nm technology used in [7], thus leading to smaller time constants and, consequently, higher power consumption.

The main contributor to these currents is the subthreshold leakage current. Gate and body leakage can be disregarded, since simulations show them to be at least two orders of magnitude smaller. Therefore, the expression for the subthreshold leakage current is [18]

(2) $$I_{DS}=I_{S}10^{\frac{V_{GS}-V_{TH0}+\lambda_{d}V_{DS}+\gamma_{d}V_{BS}}{S}}\left(1-10^{\frac{-nV_{DS}}{S}}\right)$$

where $V_{TH0}$ is the nominal threshold voltage and *n* is a fitting parameter. *S* is the subthreshold swing attending to the following equation:

(3) $$S=n\frac{k_{b}T}{q}ln\left(10\right)$$

and

(4) $$I_{S}=2n\mu C_{ox}W/L\left(\frac{k_{b}T^{2}}{q}\right)$$

where μ is the carrier mobility, $C_{ox}$ is the oxide capacitance per area, *q* is the the electron charge, $K_{b}$ is the Boltzmann constant and *T* is the temperature.

In Equation (2), the drain-induced barrier lowering (DILB), which reduces the $V_{TH}$, is modeled by $V_{TH0}-\lambda_{d}V_{DS}$ and, similarly, the body effect is taken into account through $V_{TH0}-\gamma_{d}V_{BS}$. These two effects constitute a direct mechanism to reduce the leakage currents while the transistor is turned off ($V_{GS}=0$ V).

**Figure 6:**
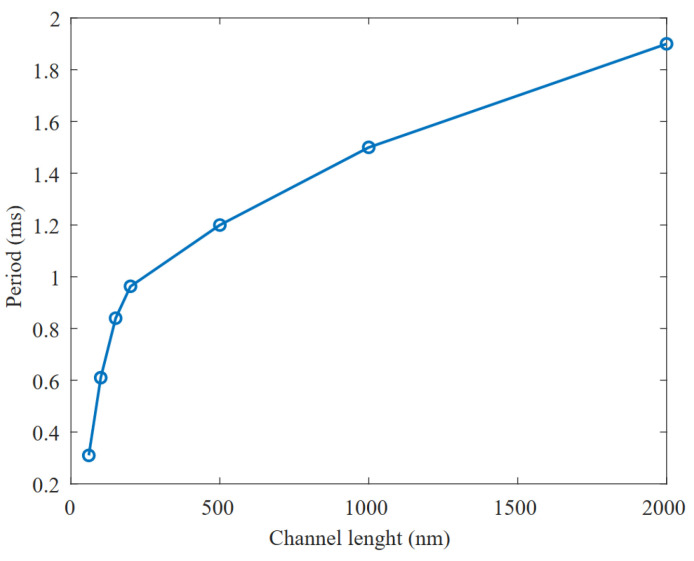
Influence of the channel length variation on the period of the generated signal.

We have taken two measures to minimize the DILB effect in Equation (2). First, it is known that the DILB effect has a greater impact on transistors with shorter channels. This is due to the depletion regions of the drain and source junctions depleting a larger fraction of the channel as the channel becomes shorter. This lowers the threshold voltage, $V_{TH}$, and causes an increase in the subthreshold leakage, which is more noticeable in smaller devices. For this reason, leakage currents can be significantly reduced by avoiding the use of minimum channel lengths. Figure 6 plots the period achieved at the output of the oscillator versus the channel length used for the thyristor transistors $M_{2,5}$ from the schematic shown in Figure 2. In our 65 nm technology, it makes sense to move from 60 nm channel length to 200 nm, as in this region any variation will provide substantial improvements. Enlarging the channel much beyond 200 nm loses effectiveness, in terms of area, in obtaining the desired delay, as the slope in Figure 6 decreases drastically.

Second, to further reduce the DILB effect, we decided to stack transistors $M_{a-d}$ in Figure 5, to reduce the $V_{DS}$ voltage. This does not require much additional area, but it has a noticeable effect on the leakage currents, as $V_{DS2,5}$ is reduced from a value close to $V_{dd}$ to just a few mV.

At the same time, to take advantage of the body effect, the bulk terminals of $M_{2,a,b}$ and $M_{5,c,d}$ were set to ground and $V_{dd}$, respectively. This increases their $V_{TH}$ and helps to further reduce the subthreshold leakage.

On the other hand, the $V_{TH0}$ in Equation (2) is another parameter that can be modified to achieve a larger delay. HVT transistors could have been used instead of standard transistors for the thyristor. This approach increases the threshold voltage by about ∼150 mV, which greatly reduces leakage currents. This reduction would translate into lower frequencies for the same area, or a reduction in capacitor size to save die area for the same target frequency. However, we ultimately rejected this option, due to the increased variability of this transistor type. Although the typical case simulations gave excellent results, we found multiple runs in the Monte Carlo simulation that did not oscillate at all. To solve this problem, the leakage currents needed to be increased, thus losing the main advantage of this technique. At the same time, this technique requires a higher minimum supply voltage (from 0.3 V to 0.7 V) for the oscillator to start operating, which can be another significant drawback in some cases.

Finally, it can be seen from Equation (1) that the leakage currents of the transistors $M_{1,6}$ should ideally be maximized, to increase the period. However, if they exceed a certain ratio to the $M_{2,5}$ leakage, the oscillator may stop oscillating. To avoid this, the currents of the transistors $M_{1,6}$ are set to 10% of the $M_{2,5}$ currents. This ensures that the VCO operates correctly even under the high process variability characteristic of thyristor-based topologies.

### 4.2. Ultra-Low Power Dissipation

Another issue with the design proposed in [7] that needs to be adjusted for our target application is the peak current. The short period of time that the transistors $M_{2,5}$ in Figure 2 are on results in a very high peak current to charge or discharge both capacitors from $V_{TH}$ to $V_{dd}$ (or GND). Since the power supply in our case comes from a weak energy harvester, a high peak current will result in an unacceptably large voltage drop. For this reason, the $M_{3}$ and $M_{4}$ gates have been biased to a voltage ($V_{bn}=0.23$ V and $V_{bp}=0.77$ V, respectively) that will not significantly affect the leakage currents that charge/discharge the capacitors, but will limit the peak currents that occur when the positive feedback is activated. In other words, the bias voltages $V_{bn}$ and $V_{bp}$ limit the maximum currents through $M_{3}$ and $M_{4}$ to a few nA, and since the leakage currents used in the thyristor are in the order of tens of pA they are not affected; thus, the frequency of the oscillator remains unchanged.

**Figure 7:**
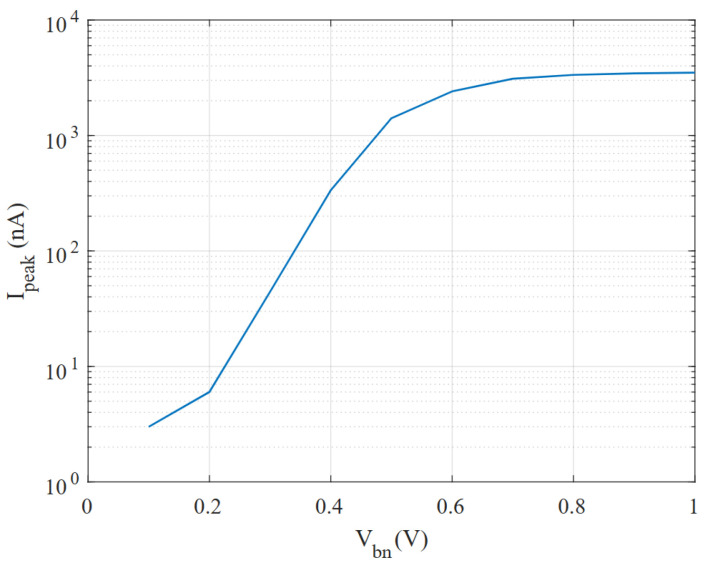
Simulation of the peak current drawn in the proposed VCO for different $V_{bn}$ values.

With the same purpose of reducing the peak power consumption, the inverters are made current-starved with the same bias voltages, $V_{bn}$ and $V_{bp}$. A simulation of how these bias voltages affect the peak currents is shown in Figure 7, to illustrate the importance of setting the correct bias for transistors $M_{3}$ and $M_{4}$ if peak currents are to be monitored. The voltages $V_{bn}$ and $V_{bp}$ are generated by a robust power-management unit present in the chip containing this oscillator.

### 4.3. Linearized Voltage-Controlled Oscillator

When it comes to dynamically changing the output frequency of the oscillator, the RC time constant of the delay element must be tuned, with respect to the input signal. We propose an approach where the capacitance of the delay element is modified to produce a quasi-linear response.

The proposed structure is shown in Figure 5. As can be seen, the capacitors used for this delay element are PMOS transistors with their source and drain terminals directly connected. CMOS transistors used as capacitors [19] are one of the most popular solutions to implement these passive components: firstly, they are available for any CMOS fabrication process without additional steps, which also translates into cheaper fabrication processes; secondly, they have the highest capacitance density of any option available in today’s processes, which is why we have chosen them to produce large time constants in the smallest possible area.

**Figure 8:**
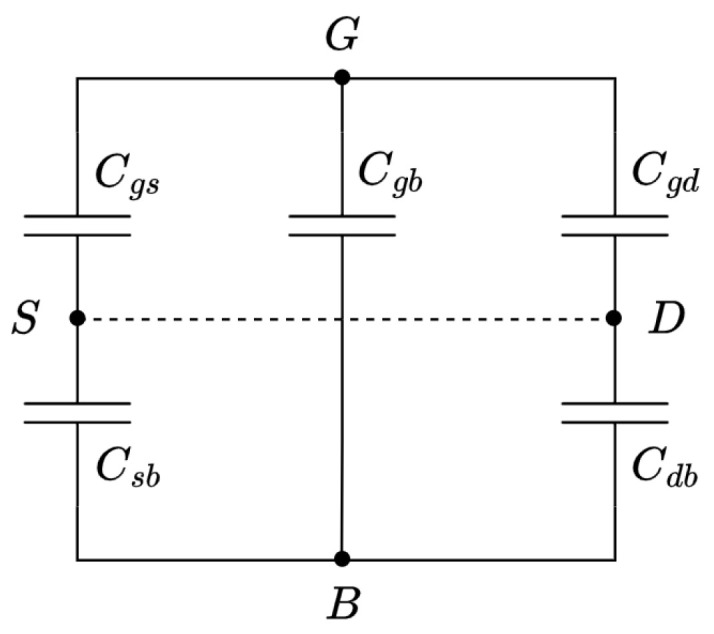
Parasitic capacitances model of an MOS transistor with source and drain connected.

The parasitic capacitance model of an MOS transistor is depicted in Figure 8. It consists mainly of the following:

- Two overlap capacitances ($C_{gs}$ and $C_{gd}$) similar to a parallel-plates capacitor and independent of the biasing voltage.
- Two junction capacitances ($C_{sb}$ and $C_{db}$) resulting from the charge accumulation fluctuation around the depletion layer. These depend on the biasing voltage.
- The gate-to-bulk capacitance ($C_{gb}$), which has two components, one bias-dependent, the channel-to-bulk capacitance, and another one independent, the gate-to-channel capacitance.

After some simplifications, the effective capacitance between gate and source–drain ($C_{eff}$) can be expressed as

(5) $$C_{eff}=\frac{\left(C_{sb}+C_{db}\right)C_{gb}}{C_{sb}+C_{db}+C_{gb}}+C_{gs}+C_{gd}$$

**Figure 9:**
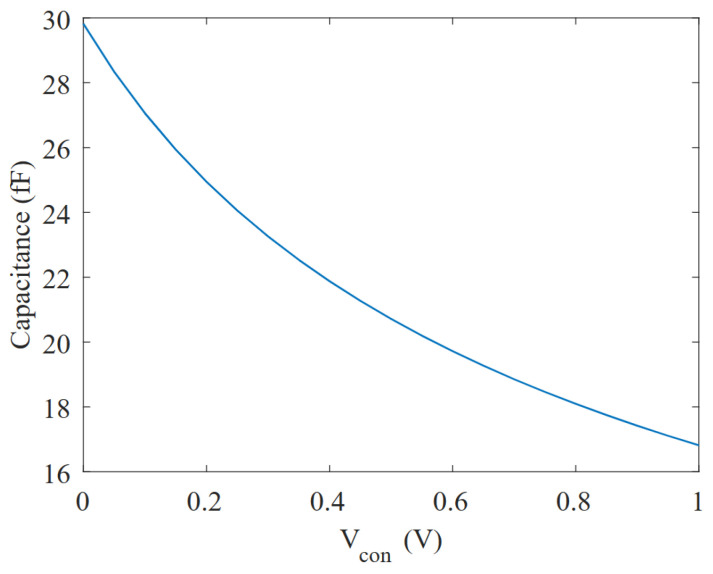
Simulation of the capacitance variation ($C_{eff}$) vs. $V_{con}$.

To vary the capacitance of the PMOS transistors ($C_{eff}$) used in the RC time constant of the VCO, the $V_{gb}$ voltage must be tuned. This directly affects the parasitic capacitances $C_{sb}$, $C_{db}$ and $C_{gb}$ by thickening or thinning their depletion layers, which act as their dielectrics. Since the only voltage-dependent term in Equation (5) consists of depletion capacitances, the capacitance $C_{eff}$, according to the depletion capacitance theory [20,21], follows a quadratic relationship with respect to the voltage (shown in Figure 9), similar to

(6) $$1/C_{eff}^{2}=K_{1}V_{con}+K_{2}$$

where $K_{1}$ and $K_{2}$ are constants that can be obtained by simulation.

Combining Equations (1) and (6), and taking into account that f=1/T, the following expression that correlates the frequency with respect to $V_{con}$ is obtained:

(7) $$f=\frac{\sqrt{K_{1}V_{con}+K_{2}}}{D}$$

where *D* stands for

(8) $$D=\frac{3V_{TH,n}}{I_{leak,M_{5}}-I_{leak,M_{6}}}$$

**Figure 10:**
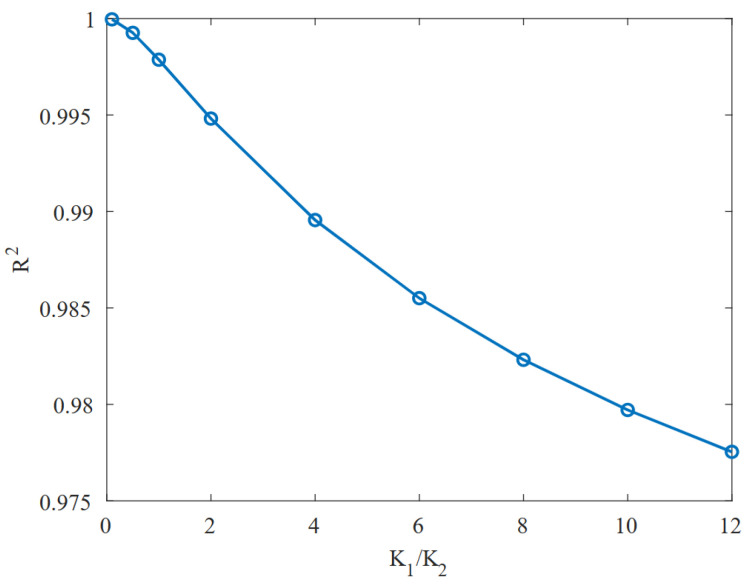
$R^{2}$ factor vs. $K_{1}/K_{2}$.

The expression given in Equation (7) can be linearized, with an $R^{2}$ factor greater than 0.99, as will be proved by experimental results in the following section, using a simple statistical regression model. The reason for this highly linear fit is the relatively small capacitance variation ratio $K_{1}/K_{2}$, which makes the square root present in Equation (7) almost flat in any available range of $V_{con}$. A graph showing how the linear regression fit evolves as a function of this ratio is shown in Figure 10. The lower $K_{1}/K_{2}$, the more linear the frequency response becomes with respect to the input voltage. However, there is a trade-off concerning the VCO gain, since a $K_{1}/K_{2}$ ratio of 0 would result in an oscillator that no longer had the ability to change its frequency, so the higher the capacitance variation ratio the higher the VCO gain. For this reason, we conducted a study to discuss the different transistors that could be used as capacitors. The most relevant parameters discussed for the selection are summarized in Table 1, namely, the $K_{1}/K_{2}$ ratio, the area and the linearity. Since for our application a linear fit greater than 0.99 ($R^{2}$) is valid, all of them provide a sufficiently linear response. Next, we focused on getting the largest possible capacitance variation ratio, which directly translates into a larger VCO gain, where LVT and core PMOS stand out with similar values, but for a small improvement in $K_{1}/K_{2}$ the LVT transistor takes up more than 40% more area than the core transistor. Consequently, the core PMOS was chosen for this design.

## 5. Results

**Figure 11:**
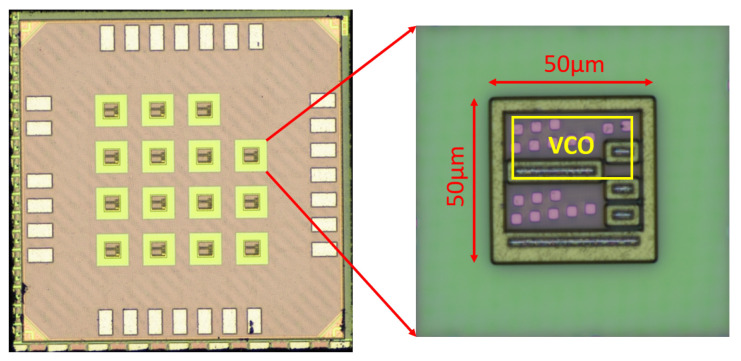
Fabricated chip.

**Figure 12:**
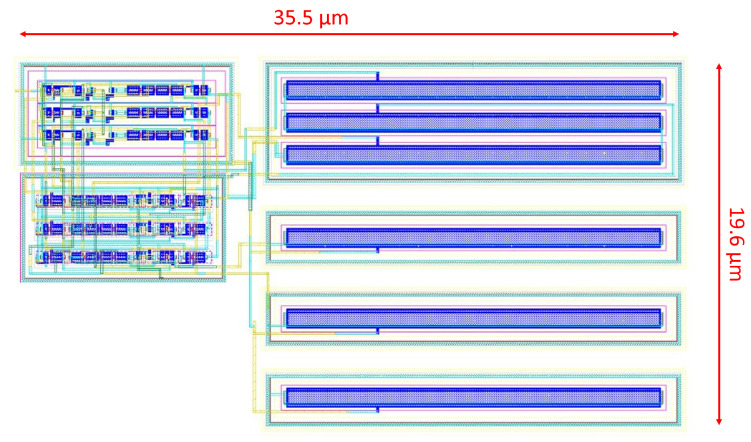
Layout of the fabricated VCO.

Figure 11 shows the fabricated CMOS silicon part of the MIT microsystem for which the oscillator was designed. The green rectangular shapes in the zoomed area are the probe pads that will later be used as the connection points for the rest of the microsystem through heterogeneous integration. The VCO, represented with a yellow box in Figure 11, occupies an area of $592\;\mu m^{2}$. The layout of the VCO is shown in Figure 12. It is worth noting that there is a space of approximately $100\;\mu m^{2}$ at the bottom-left corner for placing the biasing stage, which is part of the power-management unit (PMU) of the system, near the VCO.

Transistors $M_{a-d}$ and $M_{1,2,5,6}$ having a 200 nm/500 nm W/L ratio and the rest remaining at a minimum size of 200 nm/60 nm ratio, $C_{1}$ and $C_{2}$ were both implemented with PMOS transistors with a 700 nm/20 μm W/L ratio.

The simulation and measurements of the proposed VCO are discussed in the following subsections.

### 5.1. Simulation Results

The VCO circuit was first simulated using Virtuoso IC 6.1.7.715 from Cadence software.

**Figure 13:**
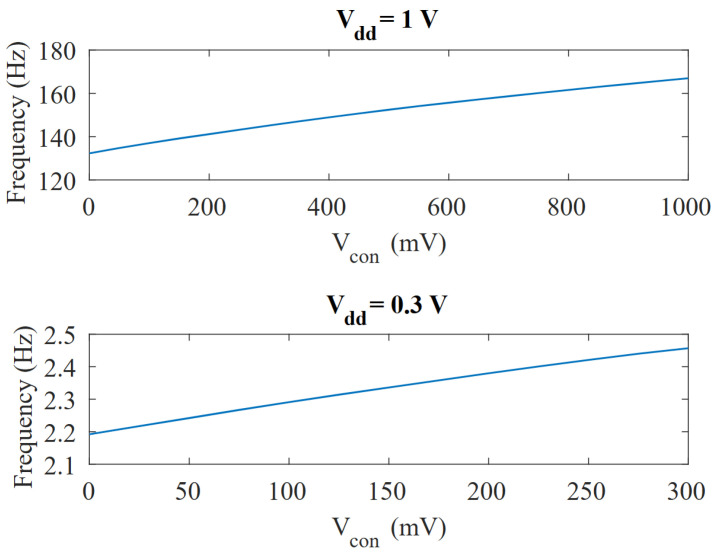
Simulation of the frequency variation vs. $V_{con}$ at $V_{dd}=1$ V (top) and $V_{dd}=0.3$ V.

The VCO response to the control voltage, $V_{con}$, at both nominal voltage (1 V) and minimum voltage (0.3 V), is shown in Figure 13. At nominal voltage, the VCO outputs a frequency that ranges from 132.4 Hz to 167 Hz, which represents a gain of ∼26%/V. At the same time, the VCO response holds good linearity, as it can be approximated by a straight line with a coefficient of determination ($R^{2}$) of 0.994.

On the other hand, when the circuit is powered at the minimum supply voltage, $V_{dd}=0.3$ V and the control voltage is swept from 0 V to $V_{dd}$, the absolute change in the capacitance is reduced but the linearity is further improved. Although the maximum frequency change also decreases (2.2 Hz to 2.46 Hz), the relative frequency shift per volt increases, due to the higher slope of the capacitance variation at lower bias voltages. As a result, when operating at 0.3 V we obtained a VCO gain of 36%/V and a coefficient of determination of 0.997. In both cases, the frequency change produced by this VCO is sufficient to perform an accurate voltage-to-frequency conversion.

Although a factor of about 1.8 was observed between the corresponding minimum and maximum capacitance values (16.5–30 fF) for the maximum and minimum $V_{con}$ possible voltages (as shown in Figure 9), for the RC time constant and, hence, the oscillation frequency experiments only a ∼26% change was observed, as shown in Figure 13.

**Figure 14:**
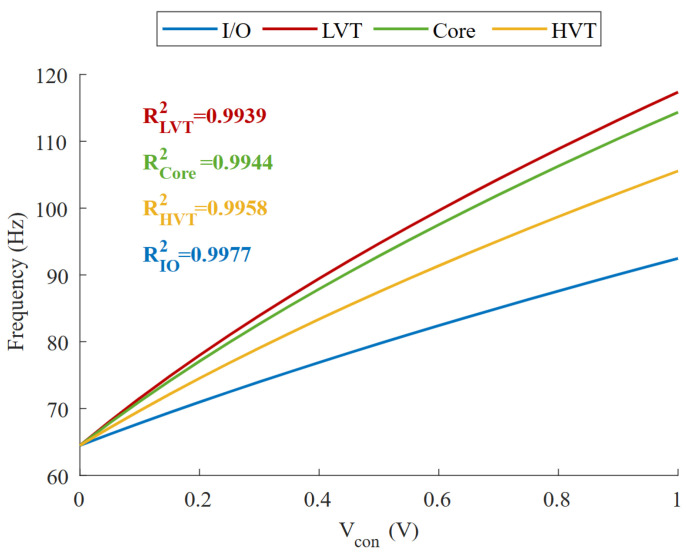
Simulation of frequency variation vs. $V_{con}$ for core, I/O, LVT and HVT transistors if both capacitances are tuned at the same time.

If double-well technology were available, both capacitors $C_{1}$ and $C_{2}$ could be tuned simultaneously by using an NMOS transistor in its own p-well, with its source and drain tied to $V_{dd}$, acting as the capacitor $C_{1}$. As a result, the same principle could be used to achieve a highly linear response with a slope of ∼2 if a higher gain was required. A theoretical model simulation, including the four available transistor options, of how the frequency changes if both capacitors could be tuned simultaneously is shown in Figure 14.

In terms of power consumption, the VCO consumes only about 200 pW at the nominal supply voltage and around 4 pW at 0.3V and remains almost invariant to the control voltage (200 pW @ 0 V and 210 pW @ 1 V). It is important to note that the peak currents have been reduced to a maximum of 11 nA at the nominal supply voltage, which is low enough to be supplied by most harvesters without any significant impact.

**Figure 15:**
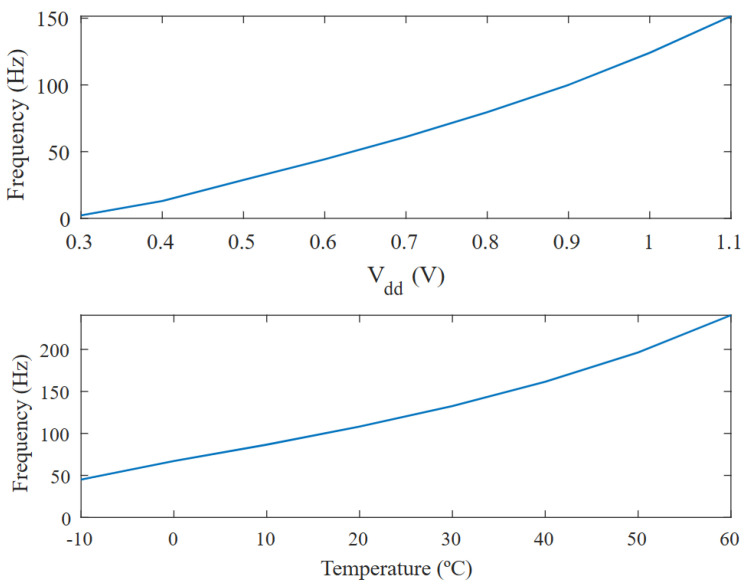
Simulation of frequency variation vs. $V_{dd}$ and temperature at $V_{con}=0$ V.

Both the voltage ($V_{dd}$) and temperature responses are shown in Figure 15. Although this VCO is intended for use in temperature-stable environments, the responses shown in Figure 15 are quite convenient if a temperature-invariant oscillator is required. If the temperature range over which the oscillator is going to operate is not too wide, the frequency variations due to temperature and supply voltage can be considered linear, making it possible to neglect the temperature effect on the frequency if the oscillator’s supply voltage comes from a specified CTAT generator that matches the observed slope. This has not yet been implemented, because in vitro operation is being considered, although it is planned for future versions of the MIT microsystem.

**Figure 16:**
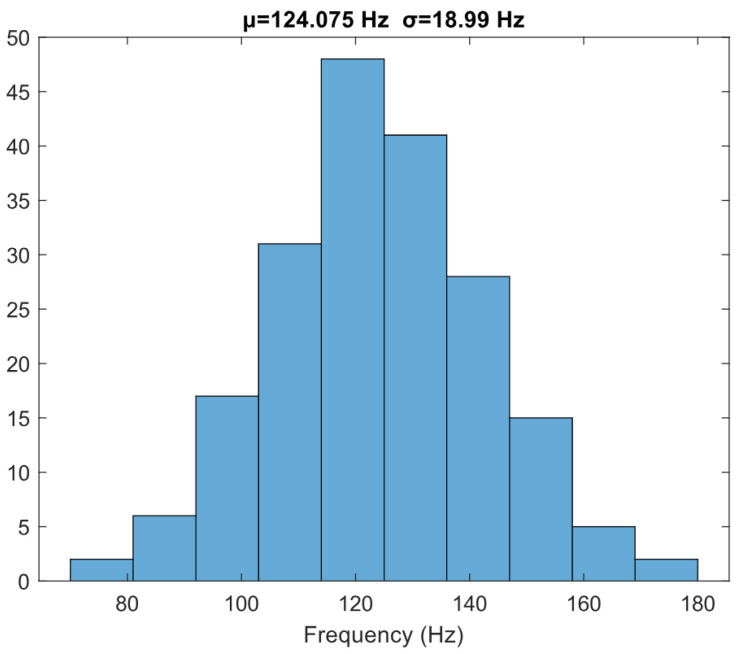
Monte Carlo frequency simulation.

The Monte Carlo was also simulated for the nominal frequency of the VCO, shown in Figure 16, giving a mean value of 124.075 Hz @ 0V and a standard deviation of 18.99 Hz (σ/μ=15.3%). The corner analysis was also simulated, and the results are collected in Table 2.

### 5.2. Measurement Results

**Figure 17:**
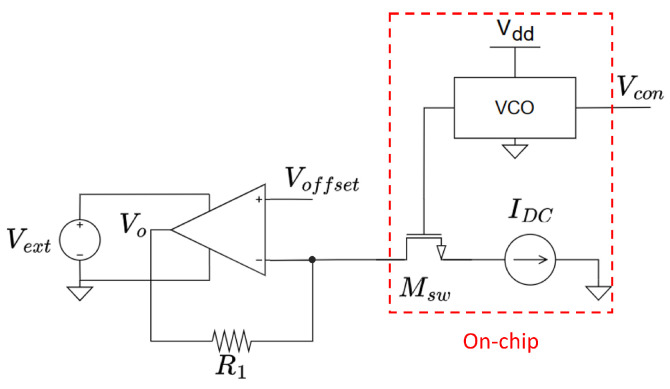
Measurement setup for the VCO.

The measurement setup is shown in Figure 17. The VCO drives a transistor acting as a switch ($M_{sw}$), which allows the current imposed by the current source, $I_{DC}$, to flow or not. These current pulses are converted into voltage pulses by a trans-impedance amplifier. The amplitude of the current pulses follows the relationship $I_{DC}\times R_{1}$, and their frequency is that of the oscillator, avoiding any possible load effect in the measurement. As also shown in Figure 17, the oscillator as well as the switching device ($M_{sw}$) and the current source $I_{DC}$ are on-chip elements, while the trans-impedance amplifier is placed off-chip, and it does not share the same power supply as the previously mentioned parts. This configuration was chosen because it accurately represents a divisible stage of the *SynCell* system.

**Figure 18:**
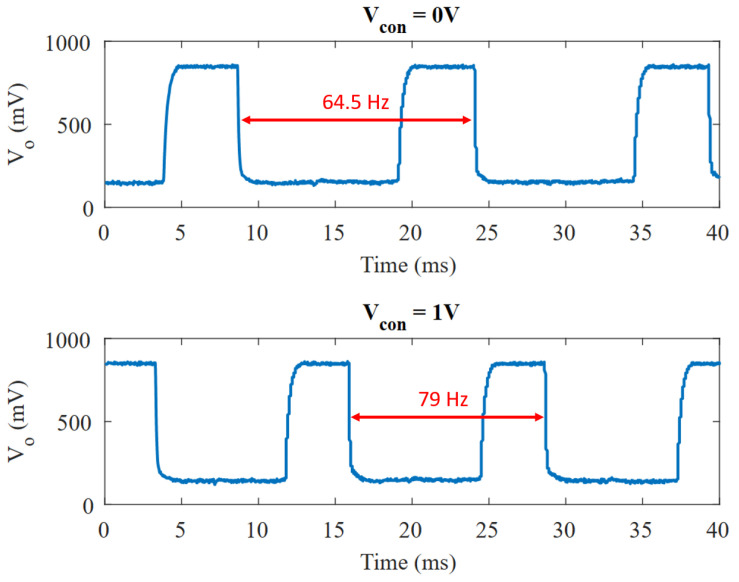
Transient measurements of the VCO at $V_{con}=0$ V and $V_{con}=1$ V.

A total of five samples from different dice were measured. Figure 18 shows the transient measurement of one sample’s output at minimum and maximum $V_{con}$ values. Table 3 collects the frequency-related data from each sample. A factor of ∼2–3x can be noticed between the simulated and the measured frequency. This also happened, to a similar extent, in [7].

**Figure 19:**
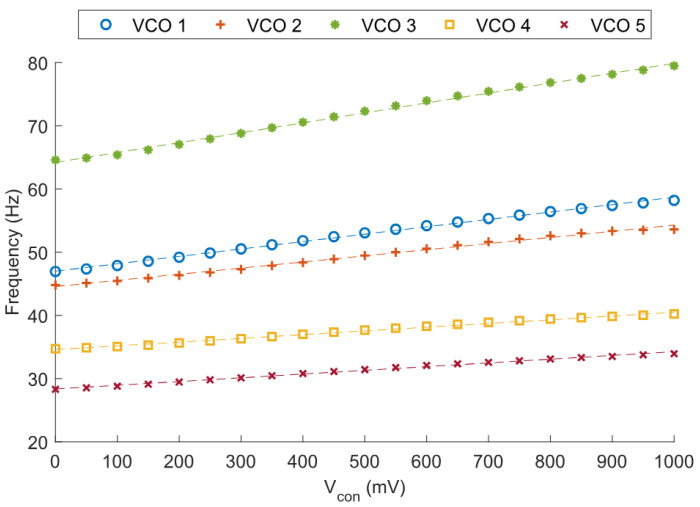
Frequency variation vs. $V_{con}$ through C-tuning for $V_{dd}=1$ V.

**Figure 20:**
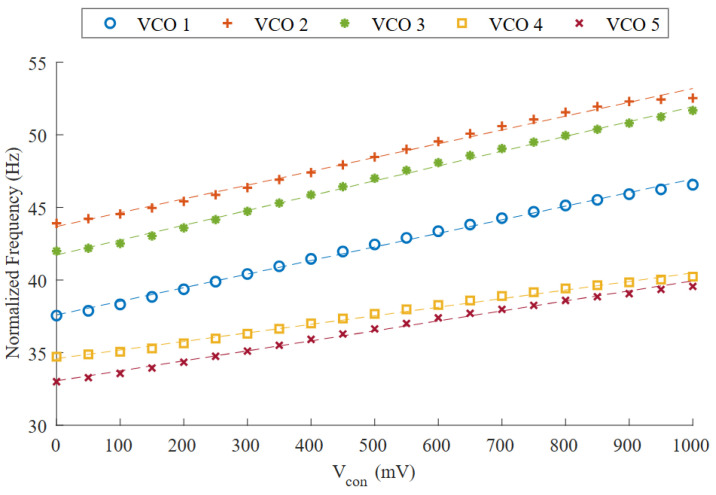
Normalized frequency variation vs. $V_{con}$ through C-tuning for $V_{dd}=1$ V.

In our case, there are a few possible reasons for this simulation-to-measurement delta. First, the models used to estimate the leakage currents may not be precise enough to determine the output frequency accurately. Another possible source of variation could be unexpected parasitic capacitance. Since the capacitors used in the circuit are very low (as low as 16 fF, in the case of $V_{con}=1$ V) any parasitic capacitance in a sensitive node, not accounted for in the post-layout simulation, can alter the output frequency. Finally, any deviation of the supply voltage from the simulation value will introduce a certain frequency delta between the measurements and the simulations. The measured VCOs are powered by a low-dropout regulator (LDO) designed to provide a 1 V output. However, process variations cause each LDO to report a different output voltage, with 0.967 V and 1.110 V being the minimum and maximum values. To account for this effect, the frequency of each sample was normalized. To perform the normalization, each LDO output was measured, and then a correction factor was applied to the measured frequencies of each sample, based on how the frequency changes with respect to the power supply. Figure 19 shows the raw VCO measurements, and Figure 20 shows the normalized responses. While there is a significant dispersion in Figure 19, presenting a σ/μ of 13.69%, after the normalization it is largely reduced to 4.65%.

The power supply voltage disparity error can be minimized with the aforementioned normalization. However, due to the process variation effect, not only does the frequency at $V_{con}=0$ V shift from one sample to another, but they also differ in their respective gains. Consequently, a 2-point calibration is required, to find out the conversion ratio between the frequency produced by the VCO and its input voltage. A straight line can be extrapolated from the two measured points and then used to model the VCO’s behavior. Because of the linear nature of the VCO, the maximum relative error committed should be small. As long as the environmental conditions, such as the temperature, remain stable, the oscillation frequency should remain invariant, so the calibration only needs to be performed once. The resolution of the digitization will eventually depend on how many pulses the counter can count in a full period, which can easily be large, due to the very slow time base generated by the oscillator.

Linearity-wise, the average coefficient of determination ($R^{2}$) measured is 0.995, with a standard deviation of 0.002 in such coefficients (μ/σ=0.2%). This slight improvement of linearity with respect to the simulations ($R^{2}$ = 0.994) is directly correlated to the slight VCO gain reduction found in the measurements. The measured frequency variation shows a change of about 22 %/V in the control voltage when working at the nominal supply (1 V), and of about 27 %/V in simulations. This difference is mainly attributable to process variation.

**Figure 21:**
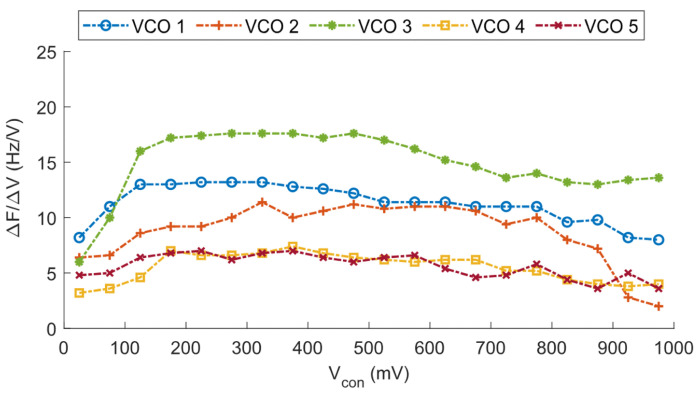
ΔF/ΔV vs. $V_{con}$ for $V_{dd}=1$ V.

To further examine the linearity of this oscillator, Figure 21 shows the derivative of the measured frequency for each measured control voltage. Ideally, a straight line would give a flat derivative. However, in our case some distortion occurs at control voltages close to the ground or $V_{dd}$, while the VCO presents a higher linearity at more centered voltages. Although the control voltage can be swept from *GND* to $V_{dd}$, we recommend restricting its range from ∼0.1 $V_{dd}$ to ∼0.9 $V_{dd}$ for a more precise linear approximation, which results in less error committed in the voltage-to-frequency conversion.

**Figure 22:**
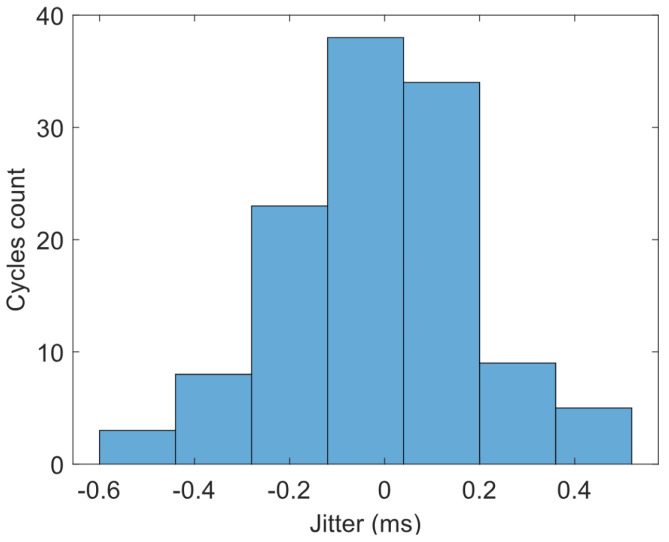
Jitter measurement of the proposed VCO at $V_{dd}=1$ V and $V_{con}=0$ V.

A common source of uncertainty for every oscillator is the jitter, measured in the time domain, or the phase noise if we consider the frequency domain. In our case, we measured the average period of one sample to be 15.4 ms at 0 V, with a standard deviation of 0.19 ms, resulting in a 1.2% relative error. The measured jitter is shown in Figure 22. To minimize the impact of jitter in our application, the system’s reader records the oscillations for a sufficient period of time (e.g., 2 s) and uses the average of the n recorded periods for voltage-to-time conversion. This approach divides the error by the number of periods counted, leading to a considerable improvement in the relative error. At the cost of slowing down the measurement process, which is not a concern for our application, it makes a reasonable conversion possible.

A comparison with the existing literature has been made in Table 4. It is important to note that the data provided for this design, such as the frequency, are the average of all the measurements carried out, instead of specific sample results.

The VCOs that we found in the literature are centered in frequency ranges going from MHz to GHz, so they consume much more power, rendering them invalid for our microsystem and, thus, comparing this work to them may be meaningless. On the other hand, we found several oscillators (without voltage control) that aim at similar area, power and frequency constraints as the one proposed here. Apart from these limitations, all of these designs have in common that they are intended to be used in temperature-stable environments, since designing a temperature-compensated structure would involve a significant area penalty, as in [10,22,23,24].

**Table 4:** Comparison of results against previous works.

Reference	This Work	[7]_*a*_	[7]_*b*_	[8]	[9]	[5]	[6]	[25]
Oscillator	subth.	subth.	subth.	subth.	subth.	gate	gate	digital
principle	leakage	leakage	leakage	leakage	leakage	leakage	leakage	with DLS
Process (nm)	65	180	180	250	350	130	55	180
$V_{dd}$ (V)	0.3–1.2	0.2–1.8	0.2–1.8	2.5	3.3	0.6	0.29	0.4
Freq. ^†^ (Hz)	43.7–53.0	10–600 × 10^3^	20	8.9	0.0308	0.09	-	-
Freq. ^‡^ (Hz)	2.2–2.46	-	20	-	-	-	0.64 *	4
Power (pW)	4 ^‡^–210 ^†^	7 × $10^{3}$–350 × $10^{6}$	3 ^‡^–230 ^†^	5.7 × $10^{3}$	1.98 × $10^{3}$	2 × $10^{3}$	4.5	3.32
Area ($\mu m^{2}$)	592	-	630	-	25,000	480	28	1600
Freq. control	yes	yes	no	no	no	no	no	no
Linear control	yes	no	no	no	no	no	no	no
FOM @$V_{dd,max}$	18.9	-	6.2	-	21.5	46.3	-	-
FOM @ $V_{dd,min}$	748.6	-	52.9	-	-	-	35,962.3 *	141.2

* Frequency and duty cycle unstable in time. Max Freq. 2.36 Hz. Min Freq. 0.16 Hz; ^†^ at nominal $V_{dd}$; ^‡^ at minimum $V_{dd}$.

The only VCO reported in Table 4 is [7]_*a*_, since it commits to ultra-low frequency, area, and power, which is the reason why it is necessary to point out some important differences between our work and [7]_*a*_ not collected in Table 4. First, our VCO allows a linear frequency control, which is the best approach for time-domain digitization, while the VCO reported in [7]_*a*_ presents an exponential frequency response. The input range in [7]_*a*_ is very limited, in some cases as constrained as just 50 mV. Our VCO, on the other hand, provides the full input voltage swing while maintaining the linearity. The current drawn by the VCO in [7]_*a*_ exhibits an undesirable exponential behavior as a function of the input voltage, deviating from the ultra-low power paradigm, but in our proposal it remains almost invariant at a very low value. Although the proposed VCO uses a smaller technology node, which should have higher process variability, the standard deviation over the mean (σ/μ) of this design is half that of [7]_*a*_, meaning that this design is less sensitive to process variations. Since the area of the VCO in [7]_*a*_ was not reported and its power consumption is taken from the given graphs, where it is not very clear how much power it requires, their well-documented only oscillator version [7]_*b*_ was also included in the comparison.

A figure of merit (FOM) has been proposed, to ease the comparison. The proposed FOM highlights those designs with low power consumption, low circuit area, low nominal frequency and large frequency variation (vs. the control voltage). Since the power consumption and the frequency are normally very dependent on the supply voltage, $V_{dd}$ has also been added to the FOM, to try to counter the effect that the different supply voltages have on each design. For those works that reported power and frequency at nominal and minimum $V_{dd}$ the FOM is given for both cases, for a fairer comparison. The proposed FOM showcases those works with better performance, in terms of the cited metrics with a higher score. The FOM is calculated with the following expression:

(9) $$FOM=\frac{\left(1+\Delta f\right)V_{dd}}{f_{0}PA}$$

This FOM has been defined to allow comparison with simple oscillators, since there are no VCOs with characteristics similar to ours. This design ranks second in this FOM, among the works cited, for minimum supply voltage. However, in this figure of merit, we had to sacrifice important features of our design, such as linearity or control range, which impaired our metrics.

## 6. Conclusions

An ultra-low-area-power-and-frequency VCO has been designed and manufactured, using 65 nm technology as a digitization method, that saves area and power at the expense of accuracy compared to more conventional analog-to-digital converters. It is based on using a sub-threshold leakage current-type delay element and provides a quasi-linear response. It minimizes leakage and limits high peak currents while allowing rail-to-rail input range swing. The oscillator occupies 592 $\mu m^{2}$, operates in the frequency range of 43.7 Hz to 53.0 Hz and consumes a maximum average power of 210 pW at nominal supply voltage. The proposed circuit shows an excellent frequency–area–power compromise not only for MIT Smart Dust SynCells but also for any biomedical application where the available space is very limited and the temperature remains stable.

## Figures and Tables

**Table 1:** PMOS transistor type comparison for their use in the VCO.

PMOS Type	$K_{1}/K_{2}$	Cap. Density (fF/$\mu m^{2}$)	Linear Fit ($R^{2}$)
Core	2.146	2.12	0.994
I/O	1.057	2.275	0.998
LVT	2.313	1.475	0.994
HVT	1.681	3.55	0.996

**Table 2:** Corner analysis simulation results at $V_{dd}=1$ V.

Corner	Tuning Range (Hz)	Gain (%/V)	$R^{2}$
TT	132.4–167.0	26.2	0.994
FF	212.0–270.1	27.4	0.988
FS	91.6–109.8	19.9	0.990
SS	85.6–120.6	40.1	0.995
SF	129.4–179.6	38.5	0.990

**Table 3:** Collected data from the five measured samples at $V_{dd}=1$ V.

VCO	Tuning Range (Hz)	Gain (%/V)	$R^{2}$	Linear Approximation
1	46.9–58.2	24.1	0.997	$f=11.7V_{con}+46.98$
2	44.8–53.7	19.9	0.993	$f=9.7V_{con}+44.57$
3	64.5–79.5	23.3	0.997	$f=15.7V_{con}+64.19$
4	34.7–40.3	16.1	0.993	$f=5.9V_{con}+34.59$
5	28.3–34.0	20.1	0.994	$f=5.9V_{con}+28.35$

## Data Availability

The original contributions presented in the study are included in the article, further inquiries can be directed to the corresponding author.
